# Emerging Roles of Sestrins in Neurodegenerative Diseases: Counteracting Oxidative Stress and Beyond

**DOI:** 10.3390/jcm8071001

**Published:** 2019-07-09

**Authors:** Shang-Der Chen, Jenq-Lin Yang, Tsu-Kung Lin, Ding-I Yang

**Affiliations:** 1 Department of Neurology, Kaohsiung Chang Gung Memorial Hospital, Kaohsiung City 83301, Taiwan; 2 Institute for Translation Research in Biomedicine; Kaohsiung Chang Gung Memorial Hospital, Kaohsiung City 83301, Taiwan; 3 College of Medicine, Chang Gung University, Taoyuan City 33302, Taiwan; 4 Institute of Brain Science, National Yang-Ming University, Taipei 11221, Taiwan; 5 Brain Research Center, National Yang-Ming University, Taipei 11221, Taiwan; 6 Taipei City Hospital, Taipei 10629, Taiwan

**Keywords:** sestrins, oxidative stress, mTOR, autophagy, neurodegenerative diseases

## Abstract

Low levels of reactive oxygen species (ROS) are critical for the operation of regular neuronal function. However, heightened oxidative stress with increased contents of oxidation markers in DNA, lipids, and proteins with compromised antioxidant capacity may play a harmful role in the brain and may be implicated in the pathophysiology of neurodegenerative diseases. Sestrins, a family of evolutionarily-conserved stress-inducible proteins, are actively regulated by assorted stresses, such as DNA damage, hypoxia, and oxidative stress. Three highly homologous genes that encode sestrin1, sestrin2, and sestrin3 proteins exist in the genomes of vertebrates. Under stressful conditions, sestrins are activated with versatile functions to cope with different types of stimuli. A growing body of evidence suggests that sestrins, especially sestrin2, can counteract oxidative stress, lessen mammalian/mechanistic target of rapamycin (mTOR) expression, and promote cell survival, thereby playing a critical role in aging-related disorders including neurodegeneration. Strategies capable of augmenting sestrin expression may; thus, facilitate cell adaptation to stressful conditions or environments through stimulation of antioxidant response and autophagy process, which may carry clinical significance in neurodegenerative diseases.

## 1. Introduction 

Reactive nitrogen species (RNS) and reactive oxygen species (ROS) are essential for maintaining the operation of regular neuronal function [1]. On the contrary, excessive RNS/ROS is proposed to play a harmful role and leads to widespread destruction of the cell membranes, DNA structures, and protein components that can, together, result in cell death. Growing evidence suggests that neurodegenerative diseases such as Alzheimer’s disease (AD), Parkinson’s disease (PD), and Huntington’s disease (HD) are all characterized by increased levels of oxidation markers, including DNA, lipids, proteins, or compromised antioxidant capacity in the brain [2,3]. Counteracting the detrimental effect of excessive RNS/ROS levels or enhancement of the antioxidant ability may be beneficial to prevent the disease development or progression in neurodegenerative disorders. 

Sestrins, a family of highly-conserved stress-inducible proteins with molecular weights ranging from 52 to 57 kDa, were discovered rather recently during probing for new stress-responsive proteins [4,5,6]. The fundamental properties of sestrins are gradually recognized in recent years [7,8]. The structure of sestrin contains three distinct functional sites, namely suppression of ROS production, regulation of mammalian/mechanistic target of rapamycin (mTOR), and leucine-binding [7,8]. The crucial roles of sestrins in various disorders including neurological diseases are emerging. In this review, we discuss recent advances in the molecular mechanisms underlying the sestrin-mediated protective effects against neuronal dysfunction in various neurological insults, especially neurodegenerative diseases. 

## 2. Energy Supply in the Brain and Oxidative Damages to the Neurons

The brain with active metabolism performs diverse signaling functions that require high energy demands, thereby resulting in overproduction of various reactive species and heightened oxidative stress. Evidence from biochemical studies suggested that a great portion of cerebral adenosine triphosphate (ATP) consumption is devoted to the process of the electrical activity of neurons [9]. Neurons are a specialized cell type in the brain exceedingly active in transmitting neuro-electrical signals through their long, extended out-reaching neurites to cope with functional needs of living organisms. High oxidative metabolism rate and relatively lower levels of antioxidant enzymes are found in neurons, making them exceptionally vulnerable to oxidative stress [10,11]. An adequate energy supply from mitochondria is; thus, indispensable for neuronal excitability and survival. Given the highly polarized neuronal structures, coordination of trafficking and anchoring of mitochondria in the axons, such that neurons can sense and respond to constantly altered energy requirements, is important for both normal function under physiological conditions and for survival under pathological conditions [12]. Furthermore, neurotransmission is an energy-demanding process that requires adequate ATP supply to support various steps, including the maintenance of ionic gradients, reversing changes in intracellular Ca^2+^ that arise from opening voltage-gated Ca^2+^ channels, as well recycling synaptic vesicles [13]. Given these reasons, it has been recognized that the brain is vulnerable to oxidative stress. Mechanistically, many factors contribute to this observation. These include redox signaling, Ca^2+^ overload-induced O_2_^−^/H_2_O_2_ generation, glutamate-induced Ca^2+^ release leading to formation of ONOO^−^ and excitotoxicity, glucose-related formation of advanced glycation end-products (AGEs), mitochondrial O_2_^-^ production, monoamine oxidase metabolism generating H_2_O_2_, neurotransmitter (e.g., dopamine) oxidation, modest or low levels of reduced glutathione (GSH), microglia-related NOX2-mediated O_2_^−^ formation, redox active transition metals (e.g., Fe^2+^ and Cu^+^), nNOS-mediated NO production, and hydroxyl radical-mediated RNA/DNA oxidation [10,14]. Thus, oxidative stress as a recognizable feature in various neurodegenerative disorders and contributes to progression of neuronal death and neural dysfunction, indicating that oxidative stress is a crucial pathogenic player in neurodegeneration [15].

**Figure 1:**
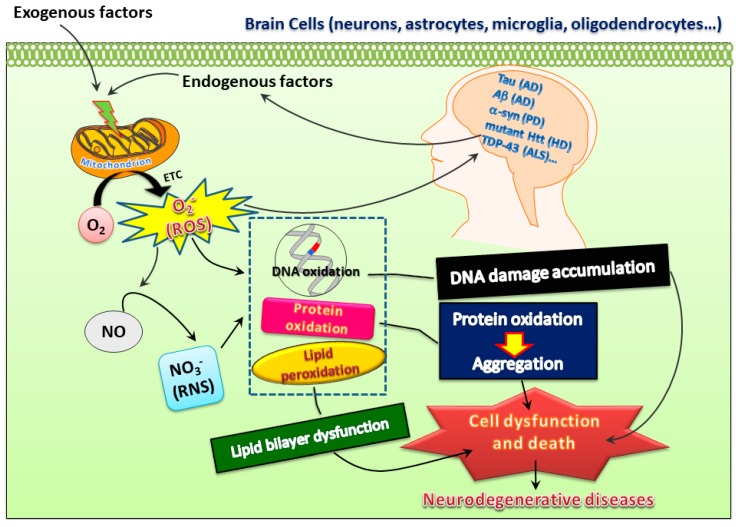
Both exogenous and endogenous factors may induce superoxide ions, reactive oxygen species (ROS), and reactive nitrogen species (RNS) to insult proteins, DNA, and lipids, which may further lead to oxidative damage and thereby create a vicious cycle to promote pathogenesis of neurodegenerative diseases. However, this also opens possibilities of developing therapeutic treatments for these diseases.

It is widely accepted that low levels of ROS and RNS are vital for maintaining neuronal function [1]. However, increased oxidative stress with heightened levels of oxidation markers in DNA, lipids, and proteins or lower antioxidant capability in the brain may play a harmful role to the organism and may be implicated in the pathophysiology of multiple neurodegenerative diseases such as AD, PD, and HD [2,3]. Various events including calcium overload, excitotoxicity, mitochondrial dysfunction, and apoptotic processes play a role in oxidative stress-mediated neurodegeneration [1]. Molecules from exogenous or endogenous origins with the ability to counteract these detrimental effects may be crucial for the development of effective treatments for neurodegenerative diseases (Figure 1).

## 3. Sestrins: An Acute Stress-Responsive Protein

**Figure 2:**
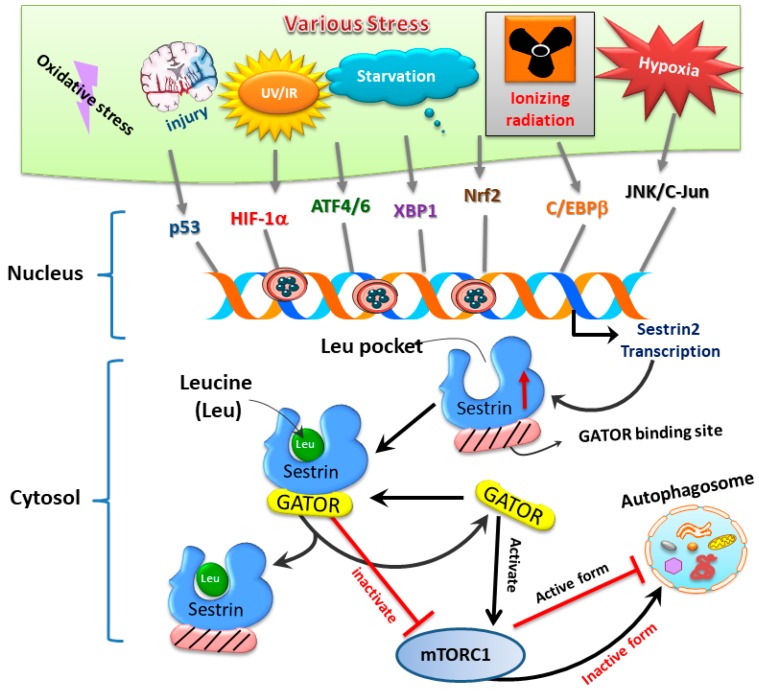
The sestrin2–GATOR interaction triggered by oxidative stress regulates mTORC1 activity and downstream autophagy homeostasis.

Sestrins, a family of stress-inducible proteins, are activated and upregulated upon exposure to hypoxia [5], oxidative stress [16], as well as genotoxic stress [6,17]. The sestrin family may possess protective functions in many physiological and pathological conditions through regulation of various mechanisms such as autophagy, endoplasmic reticulum (ER) stress, inflammation, metabolic homeostasis, and oxidative stress [18]. Three isoforms, namely sestrin1 (also known as PA26), sestrin2 (also known as Hi95), and sestrin3, are identified in mammalian cells [4,5,19]. Although the structures of sestrin1 and sestrin3 remain to be fully characterized, the crystal structure of human sestrin2 reveals two globular subdomains with distinctive functions [7]. The N-terminal domain lessens alkyl hydroperoxide radicals via its helix-turn-helix oxidoreductase motif, while the C-terminal domain adjusts this motif to physically interact with GTPase activating protein (GAP) activity towards Rags 2 (GATOR2), thereby resulting in suppression of mTOR complex 1 (mTORC1) [7]. Another functional site for leucine binding in sestrin2 was found. Binding of leucine results in a conformational adjustment and thus alters the position of the GATOR2-binding site in the C-terminal domain of sestrin2; such a leucine–sestrin2 interaction; thus, disrupts the association of sestrin2 with GATOR2 with stimulation of the mTORC1 activity [8,20]. A diagram showing stress-mediated activation of transcription factors leading to induction of sestrin and its downstream pathways regulating autophagy is depicted below in Figure 2.

Numerous factors have been identified to regulate the expression of sestrins. It was originally found that the expression of sestrin1 and sestrin2 was regulated by p53 [6], whereas the expression of sestrin3 was regulated by transcription factor forkhead box O (FOXO) [21,22]. Later, additional studies reveal several regulators crucial for the expression of sestrins under various stressful conditions; these include nuclear factor erythroid 2 like 2 (Nrf2), activating transcription factor4 (ATF4), ATF6, c-Jun N-terminal kinase (JNK)/c-Jun pathway, hypoxia-inducible factor-1α (HIF-1α), X-box-binding protein 1 (XBP1), and CCAAT/enhancer-binding protein beta (C/EBPβ) [23,24,25,26,27,28]. Together, these previous reports imply vital roles of sestrins in coping with cellular stress under different physiological and pathological conditions.

## 4. Sestrins and Oxidative Metabolism

Sestrins play a vital role in alleviating cellular oxidative damages under stressful conditions. All three family members of sestrins are activated by oxidative stress, possibly involved in the metabolism of ROS and/or its reactive metabolites [29]. Causative evidence revealed that silencing the expression of sestrin genes by shRNA can cause ROS accumulation in various cell lines [19,30], result in DNA damage and genetic instability [31,32], or lead to cell death [5,19,21,30]. In addition to mammalian cells, suppression of sestrin homologue in Drosophila, or dSesn, can also result in ROS accumulation to cause oxidative damage in skeletal muscle [33], suggesting that anti-oxidation is an evolutionarily-conserved unique property of sestrins.

Several mechanisms have been proposed to underlie the antioxidant actions of sestrins. The first mechanism involves peroxiredoxins, which are the enzymes critical for the antioxidant defense system that metabolizes peroxides in living organisms. Sestrins contain a conventional redox-active domain homologous to alkyl hydroperoxide reductase (AhpD), a crucial component of the bacterial antioxidant defense system; AhpD can catalyze the reduction to regenerate overoxidized bacterial peroxiredoxins, alkyl hydroperoxide reductase C (AhpC) [19]. Initial results indicated that purified sestrin2 protein supports ATP-dependent reduction of overoxidized peroxiredoxin-I in vitro, indicating that, unlike AhpD that is a disulfide reductase, sestrins are cysteine sulfinyl reductases [19]. Later it was reported that sestrin2, unlike sulfiredoxin, is not a reductase for cysteine sulfinic acid of peroxiredoxins [34]. Sulfiredoxin facilitates access to the peroxiredoxin active site and selectively repairs the hyperoxidized forms of typical 2-Cys peroxiredoxins in an ATP-dependent manner; however, sestrin2 lacks sequence similarity with sulfiredoxin [35]. Nevertheless, it was revealed that sestrins can activate the antioxidant transcription factor Nrf2 [36], whereas one of the target genes downstream of Nrf2 is sulfiredoxin. Thus, it is also possible that sestrins may enhance the expression of sulfiredoxin to regenerate peroxiredoxins indirectly.

**Figure 3:**
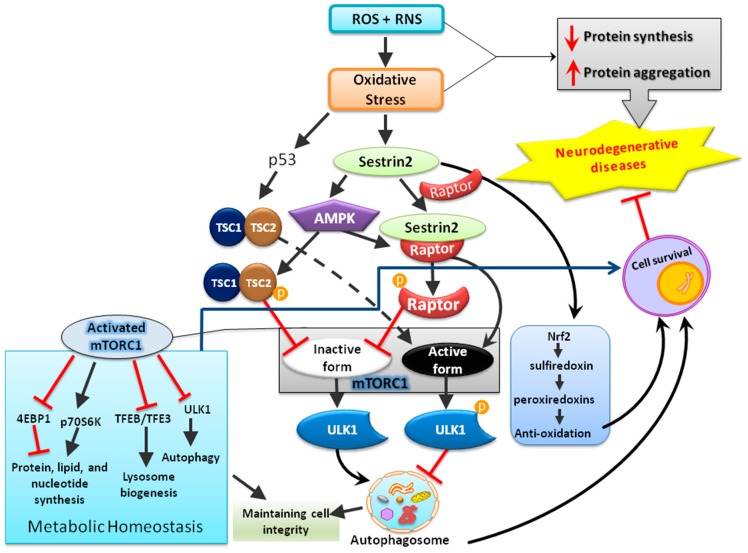
The schematic diagram illustrates that sestrin2 induced by oxidative stress plays a central role in the maintenance of cellular homeostasis by regulating multiple mediators involved in anti-oxidation, metabolism, and cellular degradation (autophagy).

In addition to regenerating overoxidized peroxiredoxins, the second anti-oxidative mechanism of sestrins contributing to redox homeostasis may be the AMP-dependent kinase/mTOR complex 1 (AMPK-mTORC1) signaling pathway that regulates autophagy, an intracellular degradation system that delivers a cytoplasmic component to the lysosomes [37]. mTOR is a serine/threonine kinase first discovered in the early 1990s as the target of rapamycin, an anti-fungal drug [38,39]. As a part of protein complexes mTORC1 and mTORC2, mTOR functions as a sensor for energy, nutrient, and redox states to regulate protein synthesis and autophagy [40,41]. Under various stressful events, such as deprivation of nutrients or deficiency of growth factors, mTOR is inhibited and autophagy is initiated [42,43]. Following a series of processes, the phagophore forms to engulf the target contents to be degraded and becomes autophagosome, which then fuses with lysosome and is termed as autophagolysosome that contains hydrolytic enzymes for the degradation of the engulfed intracellular materials from autophagosome [43]. By contrast, in the presence of nutrients or growth factors, mTOR can inhibit the kinase activity of Unc-51 like autophagy-activating kinase-1 (ULK1) and thereby suppress autophagy through phosphorylation. Under various insults, such as DNA damages and ER stress, sestrins can regulate both AMPK and mTORC1 kinase [44,45]; the former activates the process of autophagy while the latter inhibits it [46,47]. Among the intracellular organelles, mitochondria are one of the major organelles capable of ROS production and are under persistent oxidative stress from the ROS produced as metabolic byproducts during cellular respiration and oxidative phosphorylation [16,48]. Impaired mitophagy, the selective degradation of mitochondria by autophagy [49], can lead to accumulation of dysfunctional mitochondria with resultant build-up of ROS and ultimately damage macromolecules in cellular structures [16,50,51]. It was recently reported that sestrin2 can suppress sepsis through induction of mitophagy and inhibition of NLR family pyrin domain-containing 3 (NLRP3) activation in macrophages; sestrin2 enhances the autophagic efficiency by recognizing and shipping the damaged mitochondria to lysosomes for degradation [52]. Thus, activation of sestrins can eradicate the dysfunctional mitochondria through autophagy [53] as a molecular mechanism alternative to regenerating functional peroxiredoxins for the maintenance of redox balance. Notably, dysfunctional mitochondria are well-known to trigger mitochondria-dependent intrinsic apoptosis involving cytochrome c release and formation of apoptosomes. Enhancement of mitophagy by sestrins may; therefore, contribute to its anti-apoptotic actions. The potential protective mechanisms of sestrin2 in neurological disorders are shown below in Figure 3.

## 5. Sestrins in Aging-Related Clinical Conditions other than Neurodegeneration

Convincing evidence supports the contention that aging is associated with various detrimental mechanisms, such as accumulation of oxidative damages, genomic instability, loss of protein homeostasis, mitochondrial dysfunction, cellular senescence, as well as stem cell exhaustion [54]. Thus, aging is considered a risk factor for a variety of diseases in addition to neurodegeneration; these include cancer, cardiovascular diseases, type II diabetes, and stroke [54,55,56]. Inhibition of either ROS or mTORC1 can counteract aging [7] and sestrins have both characters to exert such beneficial effects [33,57]. Indeed, augmentation of sestrin expression decreases aging markers; in contrast, attenuation of sestrin expression accelerates aging processes [33,58,59,60]. Aging is accompanied with accumulation of cellular damages among various tissues in living organisms that leads ultimately to functional disruption, thereby causing disability or death. Although aging is not a specific disease entity, it is closely associated with numerous diseases [54]. 

Aging is the predestined time-dependent decline in physiological condition and a key risk factor toward cancer development. Cancer and aging share the same phenomena of time-dependent increase of cellular damages. As sestrins play a pivotal role in aging [33,61], sestrins may play crucial role in cancers. Sestrins are viewed as potential tumor suppressors. Higher sestrin2 expression is a favorable prognostic factor in patients with non-small cell lung cancer and low sestirn2 expression was associated with poor tumor differentiation, advanced tumor, node, and metastasis (TNM) stage, and lymph node metastasis [62]. In patients with colorectal cancer, it was reported that decreased expression of sestrin2 represented an unfavorable outcome [63]. Through control of mTORC1, sestrin1 is crucial to suppress the development of follicular lymphomas [64]. It was reported that the promoter area of SESN3, the sestrin3 gene, is methylated in 20% of the endometrial cancer, which denotes the character of sestrin3 as a tumor suppressor [65]. 

Hypernutrition can induce chronic activation of mTORC1 and p70S6 kinase to cause obesity and related pathology such as hepatosteatosis and insulin resistance. Activation of sestrin2 can prevent insulin resistance and fatty liver disease [66]. Induction of sestrin2 causes AMPK activation, mTORC1 inhibition, and maintenance of a high AKT activity that leads to suppression of gluconeogenesis in the liver and decrease the level of blood sugar. In contrast, sesntrin2-deficient obese mice exhibit an obvious reduction of AKT activity that causes insulin resistance and glucose overproduction [66]. Sestrin3 also carries the ability to regulate hepatic insulin sensitivity and prevents insulin resistance through maintaining AKT activity [66,67].

Previously it was demonstrated that loss of sestrins in Drosophila caused age-associated pathologies such as muscle degeneration, cardiac malfunction, and triglyceride accumulation. The cardiac malfunction includes slower heart rate and reduced heart function. The effects induced by sestrin deficiency in Drosophila were generally precluded by AICAR, the AMPK activator, and rapamycin, the mTORC1 inhibitor [33]. These findings suggest that sestrin family may play important roles in the regulation of cardiac pathophysiology [68]. 

In the endothelium, downregulation of sestrin2 increases pro-inflammatory reactions and ER stress in an AMPK-dependent way [69]. Several papers showed that sestrin2 inhibits the inflammatory pathway and lessens the extents of inflammation in macrophages, an important mediator for the formation of atherosclerosis [70,71,72].

While manipulations of sestrin expression for clinical application are still not feasible, genetic deletion of sestrins in model animals, especially in mice, has provided valuable information revealing the crucial functions of these proteins. Mice deficient in all three sestrins exhibit reduced postnatal survival associated with defective mTORC1 inactivation in multiple organs during neonatal fasting, thus revealing a non-redundant mechanism by which the sestrin family of guanine nucleotide dissociation inhibitors (GDIs) regulates the nutrient-sensing Rag GTPases to control mTORC1 signaling [73]. Sestrin2 knockout mice displayed enhanced expression of proinflammatory genes and activation of basilar membrane macrophages; these results suggest that sestrin2 plays an important role in cochlear homeostasis and immune responses to stress [74]. Other phenotypes of sestrin2 knockout mice included worsened hair cell loss in gentamicin-treated cochlear explants, implying an important role of sestrin2 in protecting hair cells against gentamicin [75]. As discussed below in the section of neurodegeneration, sestrin2 knockout mice also exhibited considerably increased late-phase neuropathic pain behavior that was associated with elevated ROS levels [76].

## 6. Potential Roles of Sestrins in Neurological Disorders and Neurodegenerative Diseases

Among the age-related disorders, neurodegenerative diseases are especially critical due to the paucity of effective treatment to date, irreversible clinical course, and accompanying heavy social economic burdens [56,77,78]. The potentially important roles of sestrins in neurological disorders have not been fully demonstrated, but they are gradually appreciated in recent years. These include seizure, neuropathic pain, ischemic stroke, and neonatal hypoxic-ischemic encephalopathy. As mentioned above, sestrins can directly suppress oxidative stress by regenerating overoxidized peroxiredoxins and indirectly mitigate oxidative stress by regulating mTOR to enhance autophagy, or more specifically mitophagy. The N-terminal domain of sestrins reduces oxidative stress through its helix-turn-helix motif, whereas the C-terminal domain of sestrins may modify this motif to adapt physical relation with GATOR2, thereby resulting in inhibition of mTORC1 [7]. However, in addition to oxidative stress, another common pathogenic mechanism of chronic neurodegenerative diseases is accumulation of aberrantly processed and/or misfolded proteins. These include amyloid-beta peptide (Aβ) and hyperphosphorylated tau protein in AD, Lewy body containing α-synuclein in PD, mutant huntingtin (Htt) in HD, TAR DNA-binding protein-43 (TDP-43) in amyotrophic lateral sclerosis (ALS), and others. Autophagy-activating drugs may; thus, open a new opportunity for the treatment of neurodegenerative diseases caused by misfolded protein toxicity [79]. The dual biological functions of sestrins, namely anti-oxidation and autophagy promotion to remove the aggregated proteins; therefore, place them in a unique position defending neurodegeneration as compared to other antioxidants.

Through studies in human patients with temporal lobe epilepsy, it was shown that sestrin3 is a regulator of a proconvulsant gene [80]. Using rat temporal lobe epilepsy as a model system, it was revealed that silencing rno-miR-155-5p can increase sestrin3 expression and reduce acute seizure activity, pathological features, and cell apoptosis [81]. Sestrins exert the beneficial effects both in central and peripheral nervous system. ROS are critical for developing and maintaining neuropathic pain; the antioxidant activity of sestrin2 lessens neuropathic pain after peripheral nerve injury [76]. These results suggest a therapeutic potential for the management of chronic neuropathic pain in human patients. Several studies have also explored the roles of sestrins in cerebral ischemia [82,83,84,85,86]. With the current knowledge of sestrins, it is presumed that sestrins possess endogenous protective properties following cerebral ischemia, which are related with blood–brain barrier (BBB) preservation, neuroprotection, and amelioration of inflammation [82]. Transient global cerebral ischemia can induce sestrin2 expression and offer a protective effect against ischemic damage in the rat hippocampal CA1 region [83]. The AMP-activated protein kinase/peroxisome proliferator-activated receptor γ coactivator-1α (AMPK/PGC-1α) pathway takes part in controlling mitochondrial biogenesis and lessens cerebral ischemia/reperfusion (I/R) damage. Downregulation of sestrins2 expression can decrease mitochondrial biogenesis, which involves the AMPK/PGC-1α pathway [84]. It was demonstrated that sestrin2 enhances AMPK phosphorylation and negatively regulates mTOR to provide neuroprotective effects in neonatal hypoxic-ischemic encephalopathy in rat pups [85]. In addition to the MAPK pathway, in neonatal rats, sestrin2 can be induced by HIF-1α to protect the BBB via reducing VEGF expression after severe hypoxia-ischemia [86]. The protective effects can be enhanced by recombinant human sestrin2 and reversed by silencing sestrin2 expression. These studies clearly demonstrated the crucial roles of sestrins as an endogenous protective mediator against various neurological disorders in the brain.

Several studies including ours explored the underlying protective mechanisms of sestrins in AD, the most common neurodegenerative disease with cognitive decline [87,88,89,90,91]. In an earlier study, it was shown that sestrin2 is one of Aβ-responsive genes in human neuroblastoma CHP134 cells [90]. We later showed that sestrin2 can be induced in primary rat cortical neurons treated with Aβ and in aged APPswe/PSEN1dE9 transgenic mice; furthermore, sestrin2 serves as an endogenous protective mediator against Aβ-induced neurotoxicity through the regulation of autophagy [92]. Presenilins are essential for regulating neurotransmitter release and mutations in the presenilin genes are the main cause of familial AD [91]. Loss of presenilin activity may impair synaptic functions leading to dementia and neurodegeneration [89]. Cells deficient in presenilin have lower levels of sestrin2 expression accompanied by mTOR dysregulation, further suggesting a crucial role of sestrin2 in AD-related conditions [88]. One previous study showed that synaptic activity, through NMDA receptor (NMDAR) signaling, boosts antioxidant defenses, reduces overoxidized peroxiredoxin, and promotes resistance to oxidative stress; more importantly, synaptic activity also upregulates sestrin2, the peroxiredoxin-activating genes via C/EBPbeta [93]. These studies reveal the critical functions of sestrin2 involving oxidative stress and nutrient-sensing through mTOR in AD-related neurodegeneration. In addition to pre-clinical investigations, a human study compared the protein concentrations of sestrin1 and sestrin2 in the serum among AD patients, mild cognitive impairment (MCI) patients, and control group. Results showed significantly higher levels of sestrin2 protein and mRNA in the AD patients compared to MCI and elderly control groups; no significant difference in sestrin1 level was observed among these study groups. These results suggested the possibility of using sestrin2 as a protein marker in AD patients [87]. In addition to AD-type dementia, sestrin2 expression is altered in the brains of subjects diagnosed with human immunodeficiency virus (HIV)-associated neurocognitive disorders. In one study, sestrin2 immunoreactivity was determined in 42 isocortex sections from HIV-1-infected subjects compared to 18 age-matched non-HIV controls and 19 advanced AD cases. With HIV infection, the sestrin2 immunoreactivity pattern shifted from neuropil predominance (N) to neuropil and neuronal-soma co-dominance (NS) and neuronal-soma predominance (S); in AD brains, sestrin2 immunoreactivity was mostly intense in the neuropil and co-localized with phosphor-Tau immunoreactivity in a subset of neurofibrillary lesions [94].

Parkinson’s disease (PD) is a common neurodegenerative disease that mainly affects the motor system with slow movement, tremor, and rigidity as clinical presentations [95]. The causes of PD remain to be fully defined but may involve both genetic and environmental factors [95]. The main pathological finding includes an abnormal α-synuclein accumulation bound to ubiquitin in the damaged central nerve cells, which is termed as Lewy body (LB) [96,97]. Immunostaining and Western blot analysis using the brains of normal controls and patients with PD and dementia with Lewy body (DLB) revealed that sestrin3 is present in brainstem-type LBs, suggesting that autophagy modulators like sesestrin3 are incorporated into LBs and that the expression of these proteins can be increased by various factors including aging [98]. Another study reported elevated expression of sestrin2 in the midbrain of patients with PD [99]. In the in vitro PD model, increases in sestrin2 expression protect against 1-methyl-4-phenylpyridinium (MPP^+^) neurotoxicity [99]. In a rotenone-induced cellular model of PD, sestrin2 can also protect dopaminergic cells against rotenone toxicity through AMPK-dependent autophagy activation [100]. 

ALS is the main type of motor neuron disease (MND) involving death of motor neurons that control voluntary movements, thereby leading to weakness in the limbs, difficulty in speech or swallowing, and respiration failure as a final cause of death [101,102]. Instead of studying nerve tissues, investigation with biopsied skeletal muscles from three ALS, three MND, and three control subjects using genome-wide gene expression analysis and RT-PCR validation showed that sestrin3 is one of the several genes that were overexpressed only in the ALS group [103]. This finding suggests that sestrins play a role in this devastating neurodegenerative disease. Further studies are needed to evaluate their potential protective roles in this disease. 

3-Nitropropionic acid (3-NP), an irreversible inhibitor of mitochondrial succinate dehydrogenase, has been used to investigate the molecular mechanisms of mitochondrial dysfunction-induced cell death and neurodegeneration in HD [104]. We have demonstrated before that brain-derived neurotrophic factor (BDNF) augments sestrin2 expression to protect neuronal damage against 3-NP-induced oxidative stress, which is via attenuation of ROS formation; moreover, BDNF induction of sestrin2 involves the pathway of NO/PKG/NF-κB [105]. Finally, in a mouse model of age-related cochlear degeneration, a significant age-related reduction in sestrin2 expression in cochlear tissues was observed, which was associated with early onset hearing loss and accelerated age-related sensory cell degeneration.

Based on the above information, a table with a list of neurological diseases in which sestrins have been investigated is shown below (Table 1). Collectively, these lines of evidence revealed that sestrins may be critical to protect various pathological conditions including neurodegeneration. Enhancing the expression levels of sestrins, especially sestrin2, may have clinical significance to prevent disease development or delay progression. With either animal models of neurological disorders or patients with neurodegenerative diseases, alteration of sestrins signaling with resultant alterations of ROS and autophagy by molecular or pharmacological approaches will advance our understanding towards these diseases and may lead the way to innovative approaches for their prevention and treatment. 

## 7. Conclusions and Future Perspectives 

The sestrin family, as critical intracellular sensors, regulate multiple processes such as autophagy, ER stress, inflammation, metabolic homeostasis, and oxidative stress. Hence, sestrins are also regarded as acute stress-responsive proteins. Emerging evidence reveals that sestrins may have important clinical functions to cope with pathological conditions resulted from various stressful conditions such as DNA damage, hypoxia, metabolic derangement, and oxidative stress. In aging-related neurological disorders, especially neurodegenerative diseases, excessive oxidative stress and autophagy play crucial roles in the pathogenesis of these diseased conditions. Sestrins, with unique functional sites to counteract ROS and mTOR regulation, are expected to play a pivotal role in neurodegenerative diseases, although, up to date, only very limited information is available to unveil their importance. In addition, most of the studies in neurological disorders focused on sestrin2, information regarding the roles of sestrin1 and sestrin3 in the nervous system is relatively scarce. Potentially unique functions of these two sestrins in neurodegeneration; therefore, warrant further investigation. Certain molecules and proteins can affect the expression levels of sestrins. It is assumed that any measure capable of enhancing sestrin expression should have clinical significance in counteracting disease-related stressful conditions. On the other hand, it should also be noted that it is currently unclear whether uncontrolled activation of sestrins may result in negative impacts; this may be significant given that sestrins are involved in autophagy regulation, whereas excessive autophagy may lead to autophagic cell death. Such a concern further reveals the critical importance for an in-depth understanding towards both the downstream and upstream regulators of sestrins. Clarification of the signal transducing pathways of sestrins would promote the discovery of innovative therapeutics for disease treatment without the potential confounding side effects, especially for the devastating neurodegenerative diseases. 

## Figures and Tables

**Table 1:** Evidence of neurological disease mechanisms related to sestrins. The symbol “**#**” indicates those reports involving human samples or clinical studies.

Neurological Diseases	Sestrins	References and Potential Mechanisms of Sestrins
Temporal lobe epilepsy	sestrin3	[80]#: to regulate proconvulsant gene network [81]: to counteract oxidative stress
Neuropathic pain	sestrin2	[76]: antioxidant activity against ROS
Cerebral ischemia	sestrin2	[83]: to regulate ribosomal protein S6 for hippocampal protection against transient global ischemia [84]: to regulate mitochondrial biogenesis via the AMPK/PGC-1α pathway for protection against focal cerebral ischemia [85]: to regulate the mTOR pathway for protection in neonatal hypoxic-ischemic encephalopathy [86]: induced by HIF-1α to protect blood-brain barrier (BBB) against severe hypoxic injury in neonatal rats
Alzheimer’s disease	sestrin2	[87]#: to increase sestrin2 protein and mRNA in the Alzheimer’s disease (AD) patients, no change in sestrin1 [88]: deficient in presenilin, mTOR dysregulation [90]: amyloid-beta peptide (Aβ)-responsive genes [92]: to regulate autophagy for protection against Aβ neurotoxicity
Parkinson’s disease	sestrin2	[99]#: Lewy body formation, p53 pathway involving ROS, and mitochondrial function [100]: neuroprotection via AMPK-dependent autophagy activation
Amyotrophic lateral sclerosis	sestrin3	[103]#: investigation with biopsied skeletal muscles
Huntington’s disease	sestrin2	[105]: Brain-derived neurotrophic factor (BDNF) induction of sestrin2 involves NO/PKG/NF-κB pathway and carries antioxidant activity
